# Spatial and temporal patterns of environmental DNA detection to inform sampling protocols in lentic and lotic systems

**DOI:** 10.1002/ece3.6014

**Published:** 2020-01-30

**Authors:** Mallory E. Bedwell, Caren S. Goldberg

**Affiliations:** ^1^ School of the Environment Washington State University Pullman WA USA

**Keywords:** environmental DNA, monitoring, *Rana boylii*, *Rana sierrae*, sampling, Sierra Nevada, study design

## Abstract

The development of efficient sampling protocols for the capture of environmental DNA (eDNA) could greatly help improve accuracy of occupancy monitoring for species that are difficult to detect. However, the process of developing a protocol in situ is complicated for rare species by the fact that animal locations are often unknown. We tested sampling designs in lake and stream systems to determine the most effective eDNA sampling protocols for two rare species: the Sierra Nevada yellow‐legged frog (*Rana sierrae*) and the foothill yellow‐legged frog (*Rana boylii*). We varied water volume, spatial sampling, and seasonal timing in lakes and streams; in lakes we also tested multiple filter types. We found that filtering 2 L versus 1 L increased the odds of detection in streams 5.42X (95% CI: 3.2–9.19X) in our protocol, from a probability of 0.51–0.85 per technical replicate. Lake sample volumes were limited by filter clogging, and we found no effect of volume or filter type. Sampling later in the season increased the odds of detection in streams by 1.96X for every 30 days (95% CI: 1.3–2.97X) but there was no effect for lakes. Spatial autocorrelation of the quantity of yellow‐legged frog eDNA captured in streams ceased between 100 and 200 m, indicating that sampling at close intervals is important.

## Graphical abstract

We developed an environmental DNA assay and a sampling protocol in situ for two rare amphibians of concern. We sampled five lakes and five streams while varying volume of water filtered, spatial sampling, and seasonal timing to increase detection efficiency. We found that filtering two liters of water, later in the season, at close spatial distances (~100 m) increased detection.

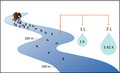

## 1 INTRODUCTION

The collection and analysis of environmental DNA (eDNA) is a high‐sensitivity approach for the detection of endangered or rare species. Environmental DNA is genetic material from environmental substrates, like soil or water, which can be collected and used to infer presence without handling or capturing the species of interest (Rees et al., 2014). The capture of DNA from a water sample indicates contemporary occupancy, as eDNA persists in aquatic environments for a few days up to a few weeks after a species is no longer present (Barnes et al., 2014; Buxton, Groombridge, & Griffiths, 2017; Dejean et al., 2011; Thomsen et al., 2012). The sensitivity of eDNA detection has been shown to be greater than traditional sampling methods in some instances (Cividae et al., 2016; Jerde, Mahon, Chadderton, & Lodge, 2011; Pilliod, Goldberg, Arkle, & Waits, 2013; Rees, Gough, Middleditch, Patmore, & Maddison, 2015; Thomsen et al., 2012), and it has a documented capability to detect low densities of animals (Dejean et al., 2011; Ficetola, Miaud, Pompanon, & Taberlet, 2008; Goldberg, Sepulveda, Ray, Baumgardt, & Waits, 2013; Rees et al., 2015). While eDNA methods are extremely sensitive, sampling design has large impacts on detection rates for target species.

The development of effective field sampling protocols is essential to the application of eDNA survey methods (Barnes et al., 2014; Eichmiller, Miller, & Sorenson, 2016; Lodge et al., 2012). The efficacy of a protocol for collecting eDNA is influenced by biotic and abiotic factors that affect how eDNA persists, which in turn alters detection (Strickler, Fremier, & Goldberg, 2015). The “ecology of eDNA” is composed of various processes and factors that affect the detection and analysis of eDNA and include origin, state, transport, and fate (Barnes & Turner, 2015). Exposure to ultraviolet radiation, warm water temperature, and acidic pH can increase degradation rates (Dejean et al., 2011; Strickler et al., 2015), likely through increased microbial activity. Research has found that there is also temporal variation in the production and detection of eDNA, with rates coinciding with different aspects of the animal's natural history, such as breeding or peak time of activity (De Souza, Godwin, Renshaw, & Larson, 2016). The size of particles containing eDNA and how they are mixed in the aquatic environment directly affect capture and the efficiency of sample volume and filter pore size (Turner et al., 2014). Additionally, the way eDNA moves in the environment, and thus how it can best be sampled, also differs by system. Environmental DNA may travel some distance from the source in flowing water (e.g., 10 km; Deiner & Altermatt, 2014), but has been estimated to decrease in concentration by half every 100 m in stream systems (Wilcox et al., 2016). Environmental DNA is more stationary in lakes (Eichmiller, Bajer, & Sorenson, 2014) and remains close (potentially < 50 m) to the source that produced it, especially when source animals are at low densities (Dunker et al., 2016). Past studies have used mesocosm experiments or placed caged animals in waterways to investigate eDNA detection (Jane et al., 2015; Wilcox et al., 2016). However, these techniques may not be available to researchers of rare and endangered species or applicable to complex real‐world conditions. Thus, the challenge is how to develop a protocol for target species in situ without direct knowledge of where the animal is.

**Figure 1:**
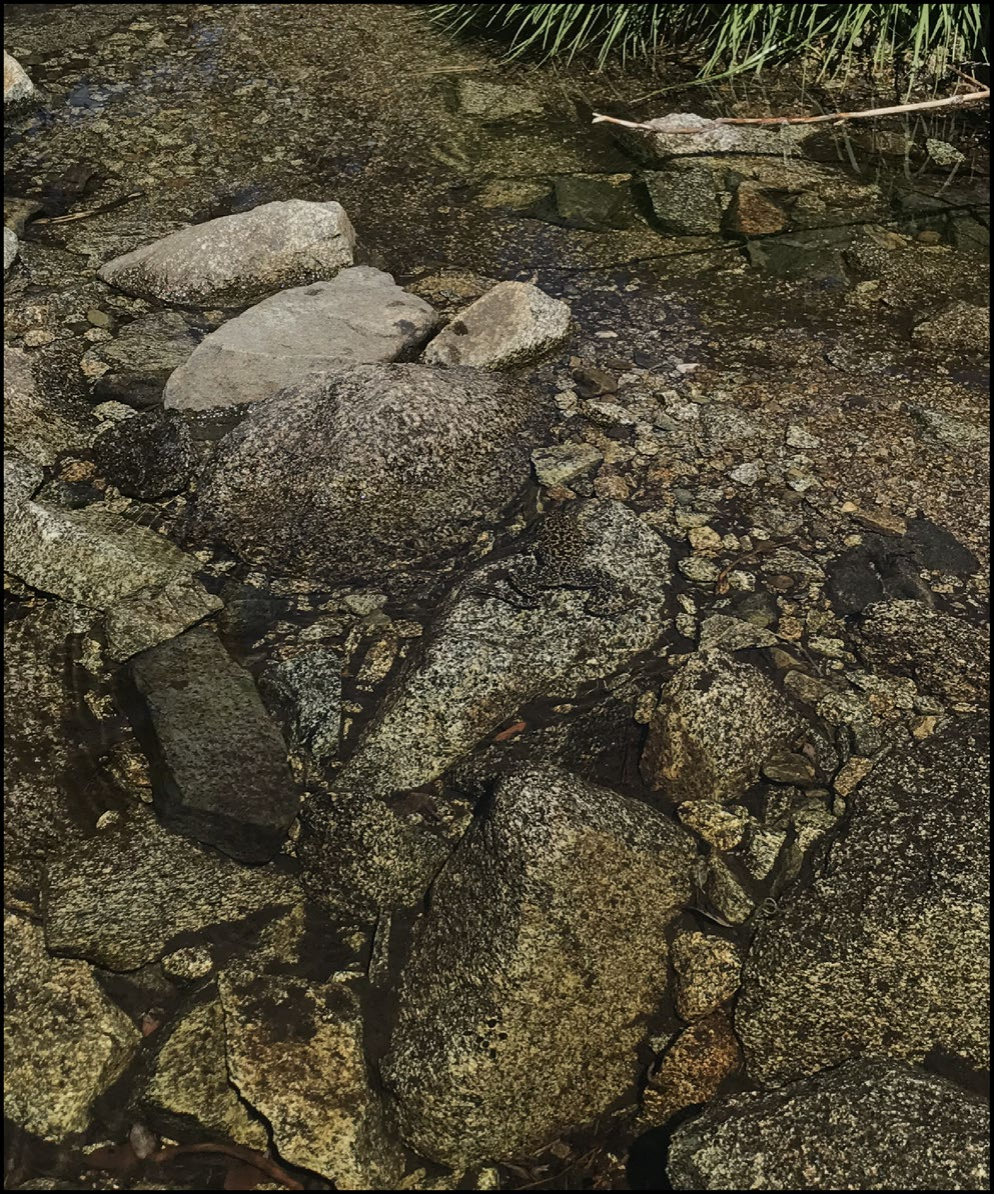
Sierra Nevada yellow‐legged frog (*Rana sierrae*) basking in a stream. Plumas National Forest, USA

Environmental DNA surveys are being increasingly utilized (Thomsen & Willerslev, 2015) and have a direct application for species conservation. Worldwide, animal populations are declining; this trend has been particularly steep for amphibians. Many amphibians spend important parts of their life cycle in water and can have low detectability (Adams et al., 2013; Green, 2003; Wake, 1991). Thus, amphibians represent an important system for the development of eDNA protocols for long‐term monitoring. Major declines have occurred for mountain‐dwelling amphibians at high elevations and streams (Stuart et al., 2004), and have been especially prevalent in the Sierra Nevada of California (Drost & Fellers, 1996; Sherman & Morton, 1993). We tested sampling designs in lake and stream systems in the Sierra Nevada to determine the most effective eDNA sampling protocols for two protected amphibian species: the Sierra Nevada yellow‐legged frog (*Rana sierrae,* Camp, 1917; Figure 1; annotated photograph Figure S1) and the foothill yellow‐legged frog (*Rana boylii,* Baird, 1856).

*Rana sierrae and R. boylii* are found in the mountains of California, USA, with *R. sierrae* primarily occupying high‐elevation lakes and outflow streams that they move to after overwintering in lakes and *R. boylii* found in streams. *Rana sierrae* has suffered large declines, including predation from introduced trout (Knapp & Matthews, 2000a, 2000b, 2000b) while *R. boylii* has been impacted by the damming of rivers and the disturbance of thermal regimes (Catenazzi & Kupferberg, 2017). Both species have experienced large population declines due to the infectious disease chytridiomycosis caused by the fungus *Batrachochytrium dendrobatidis* (Bd) (Adams, Pessier, & Briggs, 2017; Vredenburg, Knapp, Tunstall, & Briggs, 2010). *Rana sierrae* was listed as endangered under the U.S. Endangered Species Act in 2014. The federal status for *R. boylii* is currently under review by the U.S. Fish and Wildlife Service and the state of California lists five populations as endangered (central and southern Sierra Nevada and Central and South Coast) and two other populations as threated (northern Sierra Nevada and Feather River; California Department of Fish and Wildlife, 2019).

These two yellow‐legged frogs are highly aquatic, spending most of their lives in water, are challenging to find, and can overlap in streams along with their ranges. Management of these species is further complicated by their similarities in appearance, which can make identification in the field difficult. They occasionally hybridize, but hybrids do not appear to successfully reproduce (Peek et al. 2019). By relying on genetic material, instead of phenotypic characteristics, the collection of eDNA has the potential not only to more accurately monitor the distribution of these two vulnerable amphibians, but also detect the fungus Bd that has impacted both species. The systems that these frogs inhabit vary in flow, mixing, size, and substrate (Brown et al., 2019) and pose unique challenges when creating an overall sampling plan. We investigated elements of efficient eDNA sampling protocols in situ at lotic and lentic sites occupied by the two target species and analyzed the impact of volume, temporal variation, filter type, and spatial arrangement of samples on detection rate.

## 2 METHODS

### 2.1 Assay design and validation

We developed an assay for each species (*R. sierrae, R. boylii*) following the recommended three‐step design process of in silico, in vivo, and in situ phases (Goldberg et al., 2016).

#### 2.1.1 In silico

We designed quantitative PCR (qPCR) primers and probes for eDNA analysis using previously published mitochondrial sequences from the NADH dehydrogenase subunit II (ND2) gene for both *R. sierrae* (Vredenburg et al., 2007) and *R. boylii* (Lind, Spinks, Fellers, & Shaffer, 2011). Sequences for assay design were compiled from GenBank (http://www.ncbi.nlm.nih.gov) and were comprised of individuals from across the ranges of the two species. We used a total of 57 *R. sierrae* and 77 *R. boylii* sequences to create a consensus sequence for each species using Sequencher 5.4.1 (Gene Codes Corporation). We then used Primer Express (version 3.0, Applied Biosystems) to develop potential primer and probe combinations. For an assay to pass in silico validation, each of the species primers and probes was required to have at least five base pair (bp) differences total from the other yellow‐legged frog species sequence, with at least one near the 3’ end of each primer and in the center of each probe, to ensure that amplification would not occur in the opposing species (Table 1). We then tested candidate assays for specificity in Primer‐BLAST with default settings (Ye et al., 2012) to check that additional nontarget species would not amplify. If another species was found in the Primer‐BLAST search to overlap with our species of interest, we rejected the assay and tested a new primer/probe sequence.

**Table 1:** Environmental DNA primer and probe sequences for *Rana sierrae* and *Rana boylii* quantitative PCR assays. Bold indicates a base pair difference between the species

Species	Primer/probe	Sequence
R. sierrae 88 bp	RASI_F	CCTTAGCGGGCCTTCC**A**
	RASI_R	GC**A**AGTAG**A**GT**T**GCGTTTTGTTTAA**T**
	RASI‐Probe	JUN‐CCTCACAGGCTTCGC**T**CC**C**AAACTC‐QSY
R. boylii 93 bp	RABO_F	TCC**G**C**C**TCATGATC**G**AAAAC
	RABO_R	**G**GCGAAGCCTGTGAG**R**
	RABO‐Probe	6FAM‐C**C**CTTTC**CA**CAAC**C**AC**T**A‐MGB

#### 2.1.2 In vivo

We tested assays in vivo with DNA extracted from tissues of the following target and nontarget species whose distributions overlap with *R. sierrae* and *R. boylii*: Sierra Nevada yellow‐legged frog, foothill yellow‐legged frog, Cascades frog (*R. cascadae*)*,* bullfrog (*Lithobates catesbeiana*), western toad (*Anaxyrus boreas*), California red‐legged frog (*R. draytonii*), northern red‐legged frog (*R. aurora*), and Sierran treefrog (*Pseudacris sierra)*.

We analyzed samples using quantitative PCR Quantitect Multiplex PCR Mix (Qiagen, Inc, Hilden, Germany) with the concentrations recommended for multiplexing (1X QuantiTect Multiplex PCR mix, 0.2 µM of each primer, and 0.2 µM probe) on a CFX96^TM^ Real‐Time PCR Detection System (Bio‐Rad). An internal positive control (IC, Qiagen) was included in each well to test for inhibition. Total reaction volume was 15 µl and each well included 3 µl of sample. The cycling protocol began with 15 min at 95°C followed by 50 cycles of 94°C for 60 s and 62°C for 60 s. When each assay had been individually validated, we confirmed that a multiplex did not reduce reaction efficiency, sensitivity, or specificity.

A synthetic sequence (gBlock, Integrated DNA Technologies) was used to create quantitative standards for qPCR runs. We serially diluted the gBlock from 3 × 10^3^ gene copies to three gene copies per 3 µl and ran each in duplicate, except for the three copy well which was analyzed five times. The limit of detection (LOD) for each assay was determined by analyzing ten replicates of known concentrations: 20, 15, 10, 5, 3, and 1 copies/reaction. The limit of quantification for each assay was established by analyzing 24 qPCR replicates of known concentrations of 200, 100, 80, 70, 60, 50, 40, 30, 20, and 10 copies/reaction and determining the lowest concentration where the coefficient of variation (CV) was ≤0.25 (Kralik & Ricchi, 2017).

#### 2.1.3 In situ

Once the specificity each species‐specific assay had been verified, we validated assays in situ. During the summer of 2015, we collected eDNA samples at nine locations where *R. sierrae* and *R. boylii* were historically known to occur, comprised of two lakes and seven sites along streams, which were different from the locations that were later used to develop the sampling protocol. We collected water in sterile whirl‐pak bags while wearing disposable latex gloves and then vacuum‐pumped by hand (Mityvac model number MV8010) at the collection site through 0.45 µm cellulose nitrate (CN) filters in single‐use filter funnels (Nalgene, Rochester, NY). We collected four field replicates per site plus one negative control of distilled water for a total of 45 filters. Field replicates were created by filtering water collected in separate grabs from the same site. We filtered 250 ml of water per filter for ponds and 1 L of water per filter for streams. Filters were placed in 2 ml vials of molecular grade ethanol and stored at room temperature until extraction.

We extracted DNA from filter samples using the Qiashredder/DNeasy method described in Goldberg, Pilliod, Arkle, and Waits (2011). We analyzed filter samples in triplicate using the reaction optimized during in vivo validation. Environmental DNA filter sample extraction and qPCR set up was carried out in a laboratory dedicated to low‐quantity samples. Researchers are required to shower and change clothes before entering this room after being exposed to high‐quality DNA or PCR product, and no tissue samples or PCR products have been brought into this room. A negative extraction control was included with each set of extractions. A negative qPCR control was analyzed with each plate of samples.

### 2.2 Collection of eDNA samples to assess sampling design

Optimized sampling designs require information on when to sample, where to sample, and how much to sample. Sampling locations (lakes and streams) for this study were chosen based on historical presence of either yellow‐legged frog species. As eDNA behaves differently in lentic and lotic systems (Barnes & Turner, 2015), five lakes and five streams of various sizes and discharge were selected. The five montane streams varied in historical flows from 3.9 to 89.6 CFS (Wenger, Luce, Hamlet, Isaak, & Neville, 2010). Substrate at the streams was comprised of very coarse gravel, coarse gravel, small cobble, large cobble, large boulder, and bedrock (Table S2). Lake circumference ranged from 280 m to 1,280 m. Across all the lakes, most of the substrate was comprised of muck and organic detritus with some fine gravel, coarse gravel, small cobble, and large boulders (Table S3). We sampled monthly from May 16, 2016 to August 21, 2016 (Table S4). Additional filter testing was conducted once at three previous lake locations from July 11th to September 15th, 2017. Water samples were taken from the surface of the lake or stream and we attempted to avoid touching or stirring up sediment, as not to reintroduce any eDNA that may have settled. We collected samples using a latex gloved hand and sterile whirl‐pak bag. A negative control of distilled water was also filtered at each location to check for contamination from our filtration equipment or handling of samples. Once filtration was complete, the filter was folded in half using forceps that had been sterilized in a 50% bleach solution for one minute and rinsed in clean water. The filter was then placed in a coin envelope and stored in a bag filled with silica to desiccate. All filtration equipment was sterilized at the end of the day using a solution of 10% bleach and rinsed.

#### 2.2.1 Streams

**Figure 2:**
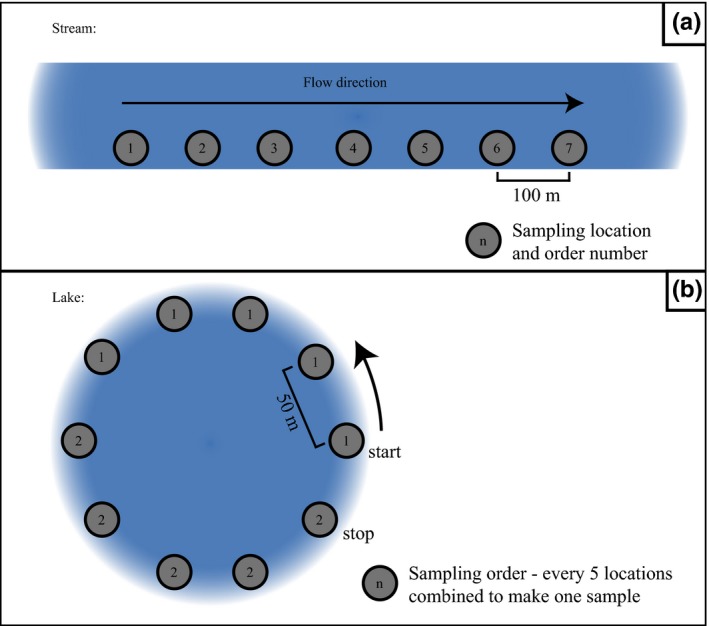
(a) Environmental DNA protocol sampling design to test effects of various sampling methods in streams. Sampling took place at seven different sites along a transect. We created two samples at each site with 2 different volumes: 1 L and 2 L. All five sites were visited at four different sampling occasions over the summer from May to August of 2016. (b) eDNA protocol sampling design to test effects of various sampling methods in lakes. Sampling consisted of combining five consecutive points, spaced 50 m apart. Each point contributed equal amounts of water to create two samples of two different target volumes: 1 L and 2 L. All five sites were visited at four different sampling occasions over the summer from May to August in 2016

To examine the most efficient spacing for stream sampling and to evaluate how far yellow‐legged frog eDNA travels in the system, we collected samples at 100 m intervals along streams known to have populations of at least one of the two target species. We took samples at seven sites spaced 100 m apart, for a total distance covered of 600 m. Water temperature and pH were recorded at each site. We took two samples at two different volumes, 1 L and 2 L, using 0.45 µm CN filters (Nalgene, Inc., item # 145‐2045) at each sampling site (Figure 2a).

#### 2.2.2 Lakes

To cover the large circumference of lakes and increase efficiency in sampling larger lake sizes, we collected water from multiple points and combined them to create a filter sample to reflect occupancy at the lake scale. We took water while standing on the shore from points spaced 50 m apart around the circumference of the lake (Figure 2b). Taking water from the shore not only is easier to attain and reduces potential for contamination, but also corresponds with the frog's littoral preferences. Water temperature and pH at every third point was noted. We then mixed a measured portion of the five consecutive water grabs in a new sterile whirl‐pak bag, thus representing a lake site. Lake sites were sampled for two different target volumes for each site: 1 L for one filter sample (200 ml from each of the five points) and 2 L for the other (400 ml from each of the five points). In practice, we stopped filtration at 40 min if the target volume was not reached due to clogging and recorded the volume filtered. If there were less than five sample points on the last portion of the lake, a greater amount of water was filtered from each sample point, so that each equally contributed to the target volumes. We discovered during the first sampling occasion that filters at several of the lake locations would clog early on while pumping water, leading to a filtration volume below the target amounts. To increase the volume filtered, we took additional samples using two filters with a larger pore size to compare against the standard 0.45 µm CN filters by changing out the filters in the same style filter funnel (Nalgene). During sampling occasion 2, we tested 5 µm polyethersulfone (PES) filters and during sampling occasion 3 and 4, we tested a 5 µm mixed cellulose ester membrane (MCE). We filtered samples at the same time to reduce differential effects of degradation.

Additional filter testing was carried out at lakes during the 2017 field season to attempt to increase volume of water filtered at lakes. We revisited three of the same eDNA protocol lakes (lakes G, I, and J) on a single sampling occasion to compare a new filter cup (Sterlitech AF045W50 disposable filter funnel) fitted with a 5 µm PES filter to the original Nalgene cups used with the 0.45 µm CN filters and 5 µm MCE filters. We collected water from the same 2016 points and combined water from the same five consecutive points. We took two samples using 0.45 µm CN, two using 5 µm MCE, and two using 5 µm PES filters in a Sterlitech filter cup from each site. Target volume filtered was 500 ml (100 ml from each point). We took a negative filter at each location using distilled water to check for contamination.

### 2.3 Analysis of eDNA samples

Each sample was analyzed in triplicate in the qPCR multiplex. A positive qPCR replicate was defined as any sample that had at least one well testing with a Cq value of 45 or below. Samples testing as inhibited were cleaned using a OneStep^TM^ PCR Inhibitor Removal Kit (Zymo, Inc., Irvine, CA). If the sample was still inhibited after this cleaning step, it was diluted by 1/10 and analyzed again. To examine the effect of volume filtered and date sampled on probability of detection, we analyzed all qPCR results using generalized linear mixed‐effect models (GLMM) in R version 3.4.2 (R Core Team, 2017. https://www.R-project.org/) using the package lme4 (Bates, Mächler, Bolker, & Walker, 2015). We analyzed streams and lakes separately. For streams, if a qPCR replicate was positive for one or both species, it was counted as just one positive detection.

#### 2.3.1 Effect of volume sampled on yellow‐legged frog detection

To examine if filtration volume influenced detection rate, each model was run using detection as a binomial response of the proportion of qPCR replicates with detections for the two volumes filtered. Location, site, and sampling occasion were used as a nested random effect. We used volume filtered as the fixed effect in the stream model and ran an intercept only model for comparison. For lake volume analysis, four models were run using the following fixed effects: volume only, filter type only, an interaction term between volume filtered and filter type, and intercept only. We defined filter type as one of the three different filters of different pore size and material that we tested. Lake volume model selection was based on model weight calculated using Akaike's Information Criterion corrected for small sample size (AICc) in the package bbmle in program R (Bolker and R Development Core Team, 2017; R Core Team, 2017).

We analyzed results from the 2017 filter comparison in lakes using the same model structure as the 2016 volume analysis. The proportion of positive qPCR replicates for each of the three filter types was the fixed effect and location and site were nested random effects. We also compared the filter type model with an intercept only model.

#### 2.3.2 Temporal variation of yellow‐legged frog detection

For analysis of the effect of seasonal timing on overall eDNA detection, we modeled the binomial response of proportion of sampling sites with detection for each species. Only filter type 1 (0.45 µm CN) was used for this analysis. We defined a positive site as one where at least two out of the six combined qPCR replicates from the two volumes filtered had positive signals (Cq ≤ 45). The date that the location was sampled was transformed into days since the first day of sampling, where 1 was May 17th. Location was used as a random effect in the sampling date model.

#### 2.3.3 Spatial patterns of yellow‐legged frog detection

We created a correlogram in GeoDa (version 1.12.1.59) to examine how eDNA signal and distance was correlated in streams. We used average starting quantity (Sq) of DNA calculated from the qPCR/liters filtered for each site at the five stream sampling locations at the four sampling occasions. Data were binned at 100 m, 200 m, 300 m, 400 m, 500 m, and 600 m distances. Moran's I was then calculated only between pairs at the same location on the same sampling occasion.

#### 2.3.4 Relationship of Bd detection to sampling protocol

In addition to the two yellow‐legged frog species, each filter sample was also analyzed for presence of the pathogenic fungus, *Batrachochytrium dendrobatidis* (Bd). We used the assay of Boyle, Boyle, Olsen, Morgan, and Hyatt (2004) and thermocycling conditions as in Kamoroff and Goldberg (2018). We then analyzed the detection results for Bd using the same mixed‐effect models for volume sampled and temporal variation.

## 3 RESULTS

### 3.1 Assay design and validation

The *R. sierrae* assay did have some late coamplification with tissue samples of the northern red‐legged frog (*R. aurora*)*;* however, the ranges of these two species do not overlap. We found that no other species coamplified with our assays. The LOD for *R. sierrae* assay was 10 copies, and the LOD for the *R. boylii* assay was five copies. The LOQ for both assays was 100 copies.

### 3.2 Analysis of eDNA samples

#### 3.2.1 Effect of volume sampled on yellow‐legged frog detection

**Figure 3:**
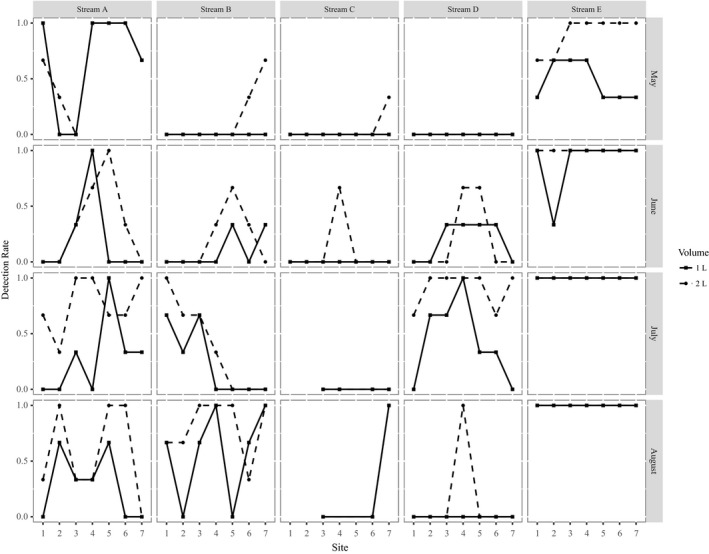
Comparing environmental DNA detection rates across sampling sites in streams for volumes of 1 L and 2 L. Sampling sites were spaced 100 m apart along a stream transect. Detection rates are the number of qPCR replicates with a yellow‐legged frog detection/total number of replicates analyzed. Streams A, B, and D were detections of *Rana boylii*, stream C were detections of *Rana sierrae* and stream E had detections of both species. Note that when only the solid line is visible that means results were identical between the volumes. Additionally, note that for stream C sites 1, 2, and 4 were dry for sampling occasion 3 and 1, 2, 4, and 5 on sampling occasion 4

Amount of water filtered at lakes varied by location and pore size (Figures S5, S6). Rate of detection varied for both volumes of water sampled at all stream locations (Figure 3). We found that filtering 2 L increased the odds of detecting yellow‐legged frogs in a qPCR replicate from streams by 5.42X (95% CI: 3.20–9.19X), with per well detection probability increasing from 0.51 to 0.85 (β = 1.7103, *p*‐value < .001; evidence weight = 1.0). For lake samples, there was no evidence that detection rate differed by volume or filter type (evidence weight for intercept only model = 0.44), including the new filter funnel tested in 2017 (evidence weight for intercept only model = 0.92).

#### 3.2.2 Temporal variation of yellow‐legged frog detection

**Figure 4:**
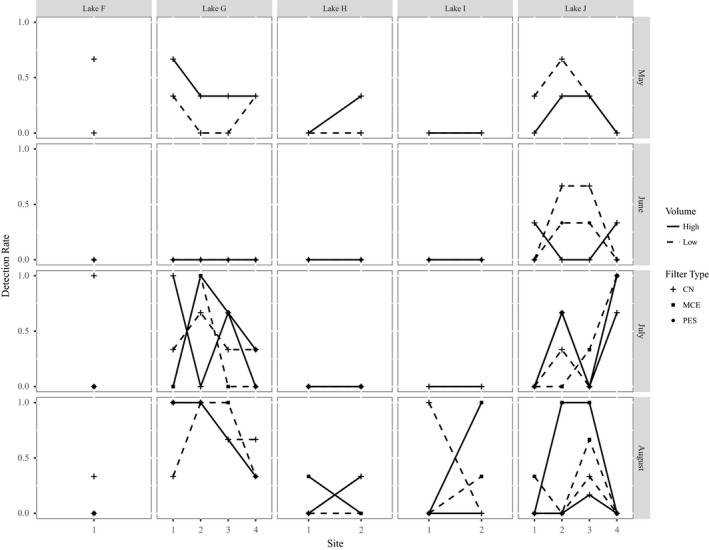
Comparing environmental DNA detection rates across sampling sites in lake protocol locations for different filter types. Detection rates are the number of qPCR replicates with a yellow‐legged frog detection/total number of replicates analyzed. All the lakes were occupied by *Rana sierrae*. Filter type 1 was the 0.45 μm CN filters and was used across all sampling occasions. On sampling occasion 2, filter type 2 was the 5 μm PES filters. For sampling occasions 3 and 4, filter type 3 was the 5 μm MCE filters

Water temperature increased over time at locations (Table S7), while pH remained similar on all sampling occasions (Table S8). Sampling later in the summer field season increased the probability of detection for streams, with the odds of a positive detection at a site on a stream increasing by 1.96X for every 30 days (95% CI: 1.30–2.97X) between May 16th and August 21st (β = 0.02247, *p*‐value = .001). There was no evidence that sampling date influenced detection at sites at lakes (*p*‐value = .206; Figure 4).

#### 3.2.3 Spatial patterns of yellow‐legged frog detection

**Figure 5:**
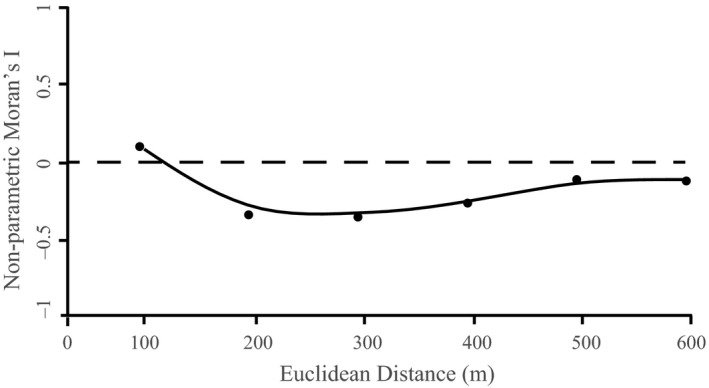
Correlogram of environmental DNA starting copy number in streams

The correlogram showed that autocorrelation reached 0 between 100 m and 200 m (Figure 5) for detections in streams. Detection of *R. sierrae* also fluctuated at lakes sites over time (Figure 4), with frogs appearing to use different sides at different sampling occasions based on proportion of positive qPCR detections.

#### 3.2.4 Relationship of Bd detection to sampling protocol

Twenty‐nine of the samples collected tested positive for Bd. We did not find evidence that Bd detection affected by volume filtered in streams (evidence weight for intercept only model = 0.36) or in lakes (evidence weight for intercept only model = 0.39). We also did not find evidence that sampling date influenced Bd detection in either streams (evidence weight for intercept only model = 0.40) or lakes (evidence weight for intercept only model = 0.23).

## 4 DISCUSSION

Using an in situ design, we found that volume, sampling date, and spatial sampling design can affect the efficacy of eDNA surveys for lotic amphibians. For lentic systems, we found that increased efficacy would be required to reach the consistency in detection rate that would be desirable for monitoring. This study shows that modification of sampling protocol can improve detection rate and can be achieved without knowledge of the species exact presence.

### 4.1 Effect of volume sampled on eDNA detection

We found that that filtering 2 L versus 1 L of water increased detection rate of yellow‐legged frogs in streams. This is expected for low‐density populations, where smaller volumes are not enough for consistent detection (Mächler, Deiner, Spahn, & Altermatt, 2016; Schultz & Lance, 2015). In systems with low overall detection rates and a low chance of clogging, filtering a larger amount of water may be the simplest way to increase detection.

In contrast to streams, increasing the volume filtered in lakes did not influence detection rates, likely due to patchiness of eDNA in lentic systems (Goldberg, Strickler, & Fremier, 2018) and lack of dispersion (Dunker et al., 2016). If eDNA remains close to the source that produced it, then capturing it also requires proximity to the source. This is made especially challenging if a species is not present in great numbers (Moyer, Diaz‐Ferguson, Hill, & Shea, 2014). Detection in lakes is thus complicated as locating the eDNA source would require many water samples spaced closely together, increasing time and effort of sampling with increasing lake size. A combination of filter material and pore size has been shown to influence detection rates (Eichmiller, Miller, & Sorensen, 2016; Turner et al., 2014) and should be evaluated to determine what works best for the size, turbidity, and species for the lake of interest. Employing larger pore sizes may help increase detection rates in environments where filters clog (Goldberg et al., 2018; Turner et al., 2014) and we did increase our volume filtered when using 5 µm MCE filters; it just did not help increase detections of our rare frog in this case. While using a larger pore size could save time and allow for more sites to be sampled, our filters slowed after filtering 1 L and usually clogged, only occasionally reaching the target volume of 2 L. Thus, time and efficiency were still an issue for the lake sites of this study. A potential solution may be to use a purpose‐designed pump that can collect a continuous sample across space (e.g., Thomas, Howard, Nguyen, Seimon, & Goldberg, 2018); this would have to be tested for efficacy in this system and ability to overcome these challenges. Water could also be collected in a transect from a boat to cover the entire lake area, if that is necessary to target the habitat used by the species of interest (Eichmiller et al., 2014).

### 4.2 Temporal variation of eDNA detection

We found that detection rate was improved later in the season in streams but did not find this relationship in lakes. For streams, this is perhaps due to the drying out of sites as the summer progressed, leading to increased population densities for these highly aquatic species. This increase appears not to be offset by the increased degradation rate expected at higher temperatures (Strickler et al., 2015). Temporal variation of eDNA detections has been found in several studies (Bista et al., 2017; Fukumoto, Ushimaru, & Minamoto, 2015; Furlan, Gleeson, Hardy, & Duncan, 2015; Laramie, Pilliod, & Goldberg, 2015), and our findings are consistent with other studies in demonstrating that there might be an optimum sampling time for species of interest that should be targeted to ensure maximum detection. For ectotherms, warmer temperatures cause increased levels of activity, which can result in higher detection rates (Buxton, Groombridge, Zakaria, & Griffiths, 2017). It has also been suggested that higher flows of streams earlier in the season may dilute eDNA available in the system (Laramie et al., 2015), and thus sampling later in the season when flows have decreased and activities have increased may enhance detection. However, detection did not increase with sampling date for lakes. This is likely because of our lower detection rates in lakes, leading to lower power to detect differences. We might expect a rise in detection later in the summer, as frogs move to overwintering habitat in lakes before snowfall. Not much is known about the movement of *R. sierrae*, but they are thought to migrate around October‐December to hibernate at the bottom of lakes that do not freeze all the way though (Vredenburg, Fellers, & Davidson, 2005). There is a balance of when to sample eDNA; detection may be improved by waiting to sample until closer to the winter, but colder temperatures can decrease activity and thus the production of eDNA.

### 4.3 Spatial patterns of eDNA detection

Environmental DNA transport has been a recurrent topic in the literature (e.g., Jerde et al., 2016; Laramie et al., 2015; Tillotson et al., 2018) and understanding how eDNA moves, especially in systems with flowing water, could help find eDNA sources and better inform sampling designs. This is especially important for the detection of rare and endangered species, where knowing exactly where populations are is critical for management. Results of this study suggest that yellow‐legged frog eDNA does not travel far in moving water, as we found that eDNA spatial autocorrelation was not detected after 100 m. This distance is consistent with other stream eDNA transport studies; Wilcox et al. (2016) found that eDNA from caged brook trout traveled between 74–145 m and that 50% of eDNA dropped out in streams every 100 m. These results indicate that sampling along a reach, rather than single‐point sampling, is important for detection of relatively sedentary species, as has been found for low‐density populations in tropical systems (Lopes et al., 2017). As detectable eDNA does not move very far from its source, point sampling could be used to indicate where within streams yellow‐legged frogs are located. If the exact location is unimportant, then it may be more efficient and cost‐effective to conduct sampling in transects or to combine multiple samples. 

For lakes, we found that eDNA signals varied from site to site and were not equal in distribution around the circumference. This uneveness is similar to what earlier studies have found (Goldberg et al., 2018; Hunter et al., 2015; Jerde et al., 2016). The eDNA signal intensity in lentic systems can correlate with high use and animal density (Eichmiller et al., 2014) and may indicate habitat preferences for location and temperature (Takahara, Minamoto, Yamanaka, Doi, & Kawabata, 2012). This preference may shift over the season, as frogs appeared to be occupying different areas of the lake at different sampling times, emphasizing the necessity to sample across space within a location.

Surveys for endangered and rare species represent a challenge for management and eDNA sampling can provide a powerful noninvasive approach to species detection. Our results indicate that effective sampling protocols for amphibians can be developed despite the limitations inherent in detecting low‐density populations that also use the terrestrial environment. For lotic systems, we recommend collecting independent replicates of 2 L samples from throughout the reach of interest, about every 100 m, using 0.45 µm CN filters. For lake systems where *R. sierrae* resides, additional work is needed to improve detection rate; an in‐line pump sampler (or similar system) that can collect replicate samples continuously across space and large volumes before clogging (Thomas et al., 2018) may be necessary for efficient detection of these rare species in lake environments. Evaluating the probability of detection through modification of sampling protocols is an important step in the development of an eDNA monitoring plan. Each of the steps in the analysis of eDNA, from capture to extraction and qPCR analysis, contribute to overall detection. Optimization of each of these components could help improve detection and broaden applications to other endangered aquatic species.

## AUTHOR CONTRIBUTIONS

M.E.B. and C.S.G. devised the study design. M.E.B. collected and processed samples. M.E.B. and C.S.G. analyzed and interpreted data. M.E.B. led on writing the manuscript. Both authors contributed to editing the drafts.

## Data Availability

eDNA results and analysis code for GLMM: Dryad https://doi.org/10.5061/dryad.2jm63xsjv.

## References

[ece36014-bib-0001] Adams, A. J. , Pessier, A. P. , & Briggs, C. J. (2017). Rapid extirpation of a North American frog coincides with an increase in fungal pathogen prevalence: Historical analysis and implications for reintroduction. Ecology and Evolution, 7, 10216–10232. 10.1002/ece3.3468 29238549 PMC5723621

[ece36014-bib-0002] Adams, M. J. , Miller, D. A. W. , Muths, E. , Corn, P. S. , Grant, E. H. C. , Bailey, L. L. , … Walls, S. C. (2013). Trends in amphibian occupancy in the United States. PLoS ONE, 8(5), e64347 10.1371/journal.pone.0064347 23717602 PMC3661441

[ece36014-bib-0003] Baird, S. F. (1856). Descriptions of new genera and species of North American Frogs In Proceedings of the academy of natural sciences of Philadelphia (Vol. 7, pp. 59–62). Philadelphia, PA: Academy of Natural Sciences of Philadelphia.

[ece36014-bib-0004] Barnes, M. A. , & Turner, C. R. (2015). The ecology of environmental DNA and implications for conservation genetics. Conservation Genetics, 17, 1–17. 10.1007/s10592-015-0775-4

[ece36014-bib-0005] Barnes, M. A. , Turner, C. R. , Jerde, C. L. , Renshaw, M. A. , Chadderton, W. L. , & Lodge, D. M. (2014). Environmental conditions influence eDNA persistence in aquatic systems. Environmental Science & Technology, 48, 1819–1827. 10.1021/es404734p 24422450

[ece36014-bib-0006] Bates, D. , Mächler, M. , Bolker, B. , & Walker, S. (2015). Fitting linear mixed‐effects models using lme4. Journal of Statistical Software, 67, 1–48. 10.18637/jss.v067.i01

[ece36014-bib-0007] Bolker, B. , & R Development Core Team (2017). bbmle: Tools for general maximum likelihood estimation. R package version 1.0. 22. https://CRAN.R-project.org/package=bbmle

[ece36014-bib-0008] Bista, I. , Carvalho, G. R. , Walsh, K. , Seymour, M. , Hajibabaei, M. , Lallias, D. , … Creer, S. (2017). Annual time‐series analysis of aqueous eDNA reveals ecologically relevant dynamics of lake ecosystem biodiversity. Nature Communications, 8, 1–11. 10.1038/ncomms14087 PMC5253663 28098255

[ece36014-bib-0009] Boyle, D. , Boyle, D. , Olsen, V. , Morgan, J. , & Hyatt, A. (2004). Rapid quantitative detection of chytridiomycosis (*Batrachochytrium dendrobatidis*) in amphibian samples using real‐time Taqman PCR assay. Diseases of Aquatic Organisms, 60, 141–148. 10.3354/dao060141 15460858

[ece36014-bib-0010] Brown, C. , Wilkinson, L. R. , Wilkinson, K. K. , Tunstall, T. , Foote, R. , Todd, B. D. , & Vredenburg, V. T. (2019). Demography, Habitat, and Movements of the Sierra Nevada yellow‐legged frog (*Rana sierrae*) in streams. Copeia, 107, 661–675. 10.1643/CE-19-196

[ece36014-bib-0011] Buxton, A. S. , Groombridge, J. J. , & Griffiths, R. A. (2017). Is the detection of aquatic environmental DNA influenced by substrate type? PLoS ONE, 12(8), e018337 10.1371/journal.pone.0183371 PMC5558973 28813525

[ece36014-bib-0012] Buxton, A. S. , Groombridge, J. J. , Zakaria, N. B. , & Griffiths, R. A. (2017). Seasonal variation in environmental DNA in relation to population size and environmental factors. Science Reports, 7, 46294 10.1038/srep46294 PMC5385492 28393885

[ece36014-bib-0013] California Department of Fish and Wildlife (2019). A status review of the foothill yellow-legged frog (*Rana boylii*) in California. Report to the Fish and Game Commission.

[ece36014-bib-0014] Camp, C. L. (1917). Notes on the systematic status of the toads and frogs of California. University of California Publications in Zoology, 17, 115–125.

[ece36014-bib-0015] Catenazzi, A. , & Kupferberg, S. J. (2017). Variation in thermal niche of a declining river‐breeding frog: From counter‐gradient responses to population distribution patterns. Freshwater Biology, 62, 1255–1265. 10.1111/fwb.12942

[ece36014-bib-0016] Civade, R. , Dejean, T. , Valentini, A. , Roset, N. , Raymond, J. C. , Bonin, A. , … Pont, D. (2016). Spatial representativeness of environmental DNA metabarcoding signal for fish biodiversity assessment in a natural freshwater system. PLoS ONE, 11(6), e0157366 10.1371/journal.pone.0157366 27359116 PMC4928825

[ece36014-bib-0017] De Souza, L. S. , Godwin, J. C. , Renshaw, M. A. , & Larson, E. (2016). Environmental DNA (eDNA) detection probability is influenced by seasonal activity of organisms. PLoS ONE, 11(10), e0165273 10.1371/journal.pone.0165273 27776150 PMC5077074

[ece36014-bib-0018] Deiner, K. , & Altermatt, F. (2014). Transport distance of invertebrate environmental DNA in a natural river. PLoS ONE, 9(2), e88786 10.1371/journal.pone.0088786 24523940 PMC3921251

[ece36014-bib-0019] Dejean, T. , Valentini, A. , Duparc, A. , Pellier‐Cuit, S. , Pompanon, F. , Taberlet, P. , & Miaud, C. (2011). Persistence of environmental DNA in freshwater ecosystems. PLoS ONE, 6(8), e23398 10.1371/journal.pone.0023398 21858099 PMC3152572

[ece36014-bib-0020] Drost, C. A. , & Fellers, G. M. (1996). Collapse of a regional frog fauna in the Yosemite area of the California Sierra Nevada, USA. Conservation Biology, 10, 414–425. 10.1046/j.1523-1739.1996.10020414.x

[ece36014-bib-0021] Dunker, K. J. , Sepulveda, A. J. , Massengill, R. L. , Olsen, J. B. , Russ, O. L. , Wenburg, J. K. , & Antonovich, A. (2016). Potential of environmental DNA to evaluate northern pike (*Esox lucius*) eradication efforts: An experimental test and case study. PLoS ONE, 11(9), e0162277 10.1371/journal.pone.0162277 27626271 PMC5023132

[ece36014-bib-0022] Eichmiller, J. J. , Bajer, P. G. , & Sorenson, P. W. (2014). The relationship between the distribution of common carp and their environmental DNA in a small lake. PLoS ONE, 9(11), e112611 10.1371/journal.pone.0112611 25383965 PMC4226586

[ece36014-bib-0023] Eichmiller, J. J. , Miller, L. M. , & Sorensen, P. W. (2016). Optimizing techniques to capture and extract environmental DNA for detection and quantification of fish. Molecular Ecology Resources, 16, 56–68. 10.1111/1755-0998.12421 25919417

[ece36014-bib-0024] Ficetola, G. F. , Miaud, C. , Pompanon, F. O. , & Taberlet, P. (2008). Species detection using environmental DNA from water samples. Biology Letters, 4, 423–425. 10.1098/rsbl.2008.0118 18400683 PMC2610135

[ece36014-bib-0026] Fukumoto, S. , Ushimaru, A. , & Minamoto, T. (2015). A basin‐scale application of environmental DNA assessment for rare endemic species and closely related exotic species in rivers: A case study of giant salamanders in Japan. Journal of Applied Ecology, 52, 358–365. 10.1111/1365-2664.12392

[ece36014-bib-0027] Furlan, E. M. , Gleeson, D. , Hardy, C. M. , & Duncan, R. P. (2015). A framework for estimating the sensitivity of eDNA surveys. Molecular Ecology Resources, 16, 641–654. 10.1111/1755-0998.12483 26536842

[ece36014-bib-0028] Goldberg, C. S. , Pilliod, D. S. , Arkle, R. S. , & Waits, L. P. (2011). Molecular detection of vertebrates in stream water: A demonstration using rocky mountain tailed frogs and Idaho giant salamanders. PLoS ONE, 6(7), e22746 10.1111/1755-0998.12643 21818382 PMC3144250

[ece36014-bib-0029] Goldberg, C. S. , Sepulveda, A. , Ray, A. , Baumgardt, J. , & Waits, L. P. (2013). Environmental DNA as a new method for early detection of New Zealand mudsnails (*Potamopyrgus antipodarum*). Freshwater Science, 32, 792–800. 10.1899/13-046.1

[ece36014-bib-0030] Goldberg, C. S. , Strickler, K. M. , & Fremier, A. K. (2018). Degradation and dispersion limit environmental DNA detection of rare amphibians in wetlands: Increasing efficacy of sampling designs. Science of the Total Environment, 633, 695–703. 10.1016/j.scitotenv.2018.02.295 29602110

[ece36014-bib-0031] Goldberg, C. S. , Turner, C. R. , Deiner, K. , Klymus, K. E. , Thomsen, P. F. , Murphy, M. A. , … Taberlet, P. (2016). Critical considerations for the application of environmental DNA methods to detect aquatic species. Methods in Ecology and Evolution, 7, 1299–1307. 10.1111/2041-210X.12595

[ece36014-bib-0032] Green, D. M. (2003). The ecology of extinction: Population fluctuation and decline in amphibians. Biological Conservation, 111, 331–343. 10.1016/S0006-3207(02)00302-6

[ece36014-bib-0033] Hunter, M. E. , Oyler‐McCance, S. J. , Dorazio, R. M. , Fike, J. A. , Smith, B. J. , Hunter, C. T. , … Hart, K. M. (2015). Environmental DNA (eDNA) sampling improves occurrence and detection estimates of invasive Burmese pythons. PLoS ONE, 10(4), e0121655 10.1371/journal.pone.0121655 25874630 PMC4398459

[ece36014-bib-0034] Jane, S. F. , Wilcox, T. M. , McKelvey, K. S. , Young, M. K. , Schwartz, M. K. , Lowe, W. H. , … Whiteley, A. R. (2015). Distance, flow and PCR inhibition: eDNA dynamics in two headwater streams. Moleclar Ecology Resources, 15, 216–227. 10.1111/1755-0998.12285 24890199

[ece36014-bib-0035] Jerde, C. L. , Mahon, A. R. , Chadderton, W. L. , & Lodge, D. M. (2011). “Sight‐unseen” detection of rare aquatic species using environmental DNA. Conservation Letters, 4, 150–157. 10.1111/j.1755-263X.2010.00158.x

[ece36014-bib-0036] Jerde, C. L. , Olds, B. P. , Shogren, A. J. , Andruszkiewicz, E. A. , Mahon, A. R. , Bolster, D. , & Tank, J. L. (2016). Influence of stream bottom substrate on retention and transport of vertebrate environmental DNA. Environmental Science Technology, 50, 8770–8779. 10.1021/acs.est.6b01761 27409250

[ece36014-bib-0037] Kamoroff, C. , & Goldberg, C. S. (2018). An issue of life or death: Using eDNA to detect viable individuals in wilderness restoration. Freshwater Science, 37, 685–696. 10.1086/699203

[ece36014-bib-0038] Knapp, R. A. , & Matthews, K. R. (2000a). Non‐native mountain fish introductions and the decline of the yellow‐legged frog from within protected areas. Conservation Biology, 14, 428–438. 10.1046/j.1523-1739.2000.99099.x

[ece36014-bib-0039] Knapp, R. A. , & Matthews, K. R. (2000b). Effects of nonnative fishes on wilderness lake ecosystems in the Sierra Nevada and recommendations for reducing impacts. Wilderness Science in a Time of Change Conference, Vol 5: Wilderness Ecosystems, Threats, and Management, 5, 312–317.

[ece36014-bib-0040] Kralik, P. , & Ricchi, M. (2017). A basic guide to real time PCR in microbial diagnostics: Definitions, parameters, and everything. Front. Microbiology, 8, 1–9. 10.3389/fmicb.2017.00108 PMC5288344 28210243

[ece36014-bib-0041] Laramie, M. B. , Pilliod, D. S. , & Goldberg, C. S. (2015). Characterizing the distribution of an endangered salmonid using environmental DNA analysis. Biological Conservation, 183, 29–37. 10.1016/j.biocon.2014.11.025

[ece36014-bib-0042] Lind, A. J. , Spinks, P. Q. , Fellers, G. M. , & Shaffer, H. B. (2011). Rangewide phylogeography and landscape genetics of the Western U.S. endemic frog *Rana boylii (Ranidae*): Implications for the conservation of frogs and rivers. Conservation Genetics, 12, 269–284. 10.1007/s10592-010-0138-0

[ece36014-bib-0043] Lodge, D. M. , Turner, C. R. , Jerde, C. L. , Barnes, M. A. , Chadderton, L. , Egan, S. P. , … Pfrender, M. E. (2012). Conservation in a cup of water: Estimating biodiversity and population abundance from environmental DNA. Molecular Ecology, 21, 2555–2558. 10.1111/j.1365-294X.2012.05600.x 22624944 PMC3412215

[ece36014-bib-0044] Lopes, C. M. , Sasso, T. , Valentini, A. , Dejean, T. , Martins, M. , Zamudio, K. R. , & Haddad, C. F. B. (2017). eDNA metabarcoding: A promising method for anuran surveys in highly diverse tropical forests. Molecular Ecology Resources, 17, 904–914. 10.1111/1755-0998.12643 27987263

[ece36014-bib-0045] Mächler, E. , Deiner, K. , Spahn, F. , & Altermatt, F. (2016). Fishing in the water: effect of sampled water volume on environmental DNA‐based detection of macroinvertebrates. Environmental Science and Technology, 50, 305–312. 10.1021/acs.est.5b04188 26560432

[ece36014-bib-0046] Moyer, G. R. , Diaz‐Ferguson, E. , Hill, J. E. , & Shea, C. (2014). Assessing environmental DNA detection in controlled lentic systems. PLoS ONE, 9(7), e103767 10.1371/journal.pone.0103767 25079969 PMC4117544

[ece36014-bib-0047] Peek, R. A. , Bedwell, M. , O'Rourke, S. M. , Goldberg, C. , Wengert, G. M. , & Miller, M. R. (2019). Hybridization between two parapatric ranid frog species in the northern Sierra Nevada, USA. Molecular Ecology, 28, 4636–4647. 10.1111/mec.15236 31495012

[ece36014-bib-0048] Pilliod, D. S. , Goldberg, C. S. , Arkle, R. S. , & Waits, L. P. (2013). Estimating occupancy and abundance of stream amphibians using environmental DNA from filtered water samples. Canadian Journal of Fisheries and Aquatic Sciences, 70, 1123–1130. 10.1139/cjfas-2013-0047

[ece36014-bib-0049] R Core Team (2017). R: A language and environment for statistical computing. Vienna, Austria: R Foundation for Statistical Computing https://www.R-project.org/

[ece36014-bib-0050] Rees, H. C. , Bishop, K. , Middleditch, D. J. , Patmore, J. R. M. , Maddison, B. C. , & Gough, K. C. (2014). The application of eDNA for monitoring of the Great Crested Newt in the UK. Ecology and Evolution, 4, 4023–4032. 10.1002/ece3.1272 25505530 PMC4242556

[ece36014-bib-0051] Rees, H. C. , Gough, K. C. , Middleditch, D. J. , Patmore, J. R. M. , & Maddison, B. C. (2015). Applications and limitations of measuring environmental DNA as indicators of the presence of aquatic animals. Journal of Applied Ecology, 52, 827–831. 10.1111/1365-2664.12467

[ece36014-bib-0052] Schultz, M. T. , & Lance, R. F. (2015). Modeling the sensitivity of field surveys for detection of environmental DNA (eDNA). PLoS ONE, 10(10), e0141503 10.1371/journal.pone.0141503 26509674 PMC4624909

[ece36014-bib-0053] Sherman, C. K. , & Morton, M. L. (1993). Population declines of Yosemite toads in the eastern Sierra Nevada of California. Journal of Herpetology, 27, 186–198. 10.2307/1564935

[ece36014-bib-0054] Strickler, K. M. , Fremier, A. K. , & Goldberg, C. S. (2015). Quantifying effects of UV‐B, temperature, and pH on eDNA degradation in aquatic microcosms. Biological Conservation, 183, 85–92. 10.1016/j.biocon.2014.11.038

[ece36014-bib-0055] Stuart, S. N. , Chanson, J. S. , Cox, N. A. , Young, B. E. , Rodrigues, A. S. L. , Fischman, D. L. , & Waller, R. W. (2004). Status and trends of amphibian declines and extinctions worldwide. Science, 306, 1783–1786. 10.1126/science.1103538 15486254

[ece36014-bib-0056] Takahara, T. , Minamoto, T. , Yamanaka, H. , Doi, H. , & Kawabata, Z. (2012). Estimation of fish biomass using environmental DNA. PLoS ONE, 7(4), e35868 10.1371/journal.pone.0035868 22563411 PMC3338542

[ece36014-bib-0057] Thomas, A. C. , Howard, J. , Nguyen, P. , Seimon, T. A. , & Goldberg, C. S. (2018). ANDe^TM^: A fully‐integrated environmental DNA sampling system. Methods in Ecology and Evolution, 10.1111/2041-210X.12994

[ece36014-bib-0058] Thomsen, P. F. , Kielgast, J. , Iversen, L. L. , Wiuf, C. , Rasmussen, M. , Gilbert, M. T. P. , … Willerslev, E. (2012). Monitoring endangered freshwater biodiversity using environmental DNA. Molecular Ecology, 21, 2565–2573. 10.1111/j.1365-294X.2011.05418.x 22151771

[ece36014-bib-0059] Thomsen, P. F. , & Willerslev, E. (2015). Environmental DNA – An emerging tool in conservation for monitoring past and present biodiversity. Biological Conservation, 183, 4–18. 10.1016/j.biocon.2014.11.019

[ece36014-bib-0060] Tillotson, M. D. , Kelly, R. P. , Duda, J. J. , Hoy, M. , Kralj, J. , & Quinn, T. P. (2018). Concentrations of environmental DNA (eDNA) reflect spawning salmon abundance at fine spatial and temporal scales. Biological Conservation, 220, 1–11. 10.1016/j.biocon.2018.01.030

[ece36014-bib-0061] Turner, C. R. , Barnes, M. A. , Xu, C. C. Y. , Jones, S. E. , Jerde, C. L. , & Lodge, D. M. (2014). Particle size distribution and optimal capture of aqueous macrobial eDNA. Methods in Ecology and Evolution, 5, 676–684. 10.1111/2041-210X.12206

[ece36014-bib-0062] Vredenburg, V. T. , Bingham, R. , Knapp, R. , Morgan, J. A. T. , Moritz, C. , & Wake, D. (2007). Concordant molecular and phenotypic data delineate new taxonomy and conservation priorities for the endangered mountain yellow‐legged frog. Journal of Zoology, 271, 361–374. 10.1111/j.1469-7998.2006.00258.x

[ece36014-bib-0063] Vredenburg, V. T. , Fellers, G. M. , & Davidson, C. (2005). Amphibian declines: Conservation status of United States species In Lannoo M. J. (Ed.), Amphibian declines: Conservation status of United States species (pp. 563–566). Berkeley, CA: University of California Press.

[ece36014-bib-0064] Vredenburg, V. T. , Knapp, R. A. , Tunstall, T. S. , & Briggs, C. J. (2010). Dynamics of an emerging disease drive large‐scale amphibian population extinctions. Proceedings of the National Academy of Science, 107, 9689–9694. 10.1073/pnas.0914111107 PMC2906868 20457913

[ece36014-bib-0065] Wake, D. B. (1991). Declining amphibian populations. Science, 253, 860 10.1126/science.253.5022.860 17751819

[ece36014-bib-0066] Wegner, S. J. , Luce, C. H. , Hamlet, A. F. , Isaak, D. J. , & Neville, H. M. (2010). Macrosacle hydrologic modeling of ecologically relevant flow metrics. Water Resources Research, 46, W09513 10.1029/2009WR008839

[ece36014-bib-0067] Wilcox, T. M. , McKelvey, K. S. , Young, M. K. , Sepulveda, A. J. , Shepard, B. B. , Jane, S. F. , … Schwartz, M. K. (2016). Understanding environmental DNA detection probabilities: A case study using a stream‐dwelling char *Salvelinus fontinalis* . Biological Conservation, 194, 209–216. 10.1016/j.biocon.2015.12.023

[ece36014-bib-0068] Ye, J. , Coulouris, G. , Zaretskaya, I. , Cutcutache, I. , Rozen, S. , & Madden, T. L. (2012). Primer‐BLAST: A tool to design target‐specific primers for polymerase chain reaction. BioMed Central, 10.1186/1471-2105-13 PMC3412702 22708584

