# Supplementary figures and images for: Spatial and temporal patterns of environmental DNA detection to inform sampling protocols in lentic and lotic systems
Source: Ecol Evol. 2020 Jan 30;10(3):1602–12. doi: 10.1002/ece3.6014 (PMC7029092; doi:10.1002/ece3.6014)

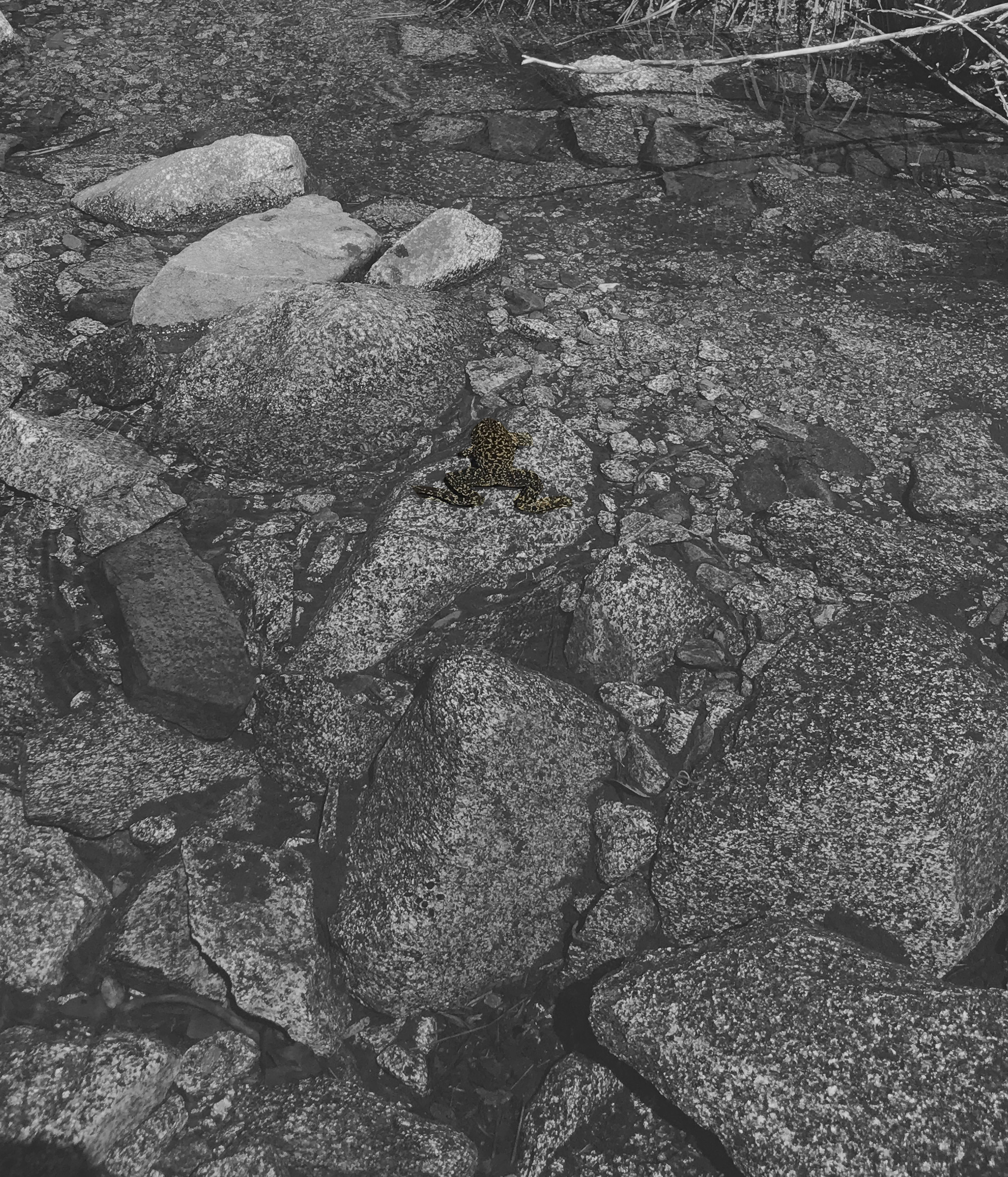

Supplement: Supplementary file 1 [file ECE3-10-1602-s001.png]
