# Supplementary material for: Spatial and temporal patterns of environmental DNA detection to inform sampling protocols in lentic and lotic systems
Source: Ecol Evol. 2020 Jan 30;10(3):1602–12. doi: 10.1002/ece3.6014 (PMC7029092; doi:10.1002/ece3.6014)
Supplement: Supplementary file 2 [file ECE3-10-1602-s002.docx]

**Appendix 2**

| eDNA Protocol Location | Historic Flow (CFS) | Substrate Types |
| --- | --- | --- |
| Stream A | 3.9 | small cobble, large cobble, large boulder, bedrock |
| Stream B | 8.2 | small cobble, large cobble, bedrock |
| Stream C | 9.3 | - |
| Stream D | 89.6 | coarse gravel, small cobble, large cobble, bedrock |
| Stream E | 49.4 | very coarse gravel, small gravel, large boulder |

| eDNA Protocol Location | Circumference (m) | Substrate Type |
| --- | --- | --- |
| Lake F | 280 | muck and organic detritus |
| Lake G | 1280 | muck and organic detritus, small cobble, large boulder |
| Lake H | 470 | muck and organic detritus |
| Lake I | 800 | muck and organic detritus, fine gravel, coarse gravel, large boulder |
| Lake J | 840 | muck and organic detritus, large boulder |

**Appendix 3**

| eDNA Protocol Location | Number of Sample Points | Total Number of Sites | Species Present | Sampling Occasion 1 | Sampling Occasion 2 | Sampling Occasion 3 | Sampling Occasion 4 |
| --- | --- | --- | --- | --- | --- | --- | --- |
| Stream A | 7 | 7 | *R. boylii* | 5/18/2016 | 6/19/2016 | 7/19/2016 | 8/18/2016 |
| Stream B | 7 | 7 | *R. boylii* | 5/24/2016 | 6/22/2016 | 7/22/2016 | 8/19/2016 |
| Stream C | 7 | 7 | *R. sierrae* | 5/16/2016 | 6/14/2016 | 7/20/2016 | 8/16/2016 |
| Stream D | 7 | 7 | *R. boylii* | 5/25/2016 | 6/20/2016 | 7/22/2016 | 8/21/2016 |
| Stream E | 7 | 7 | *R. boylii,*  *R. sierrae* | 5/20/2016 | 6/16/2016 | 7/19/2016 | 8/16/2016 |
| Lake F | 6 | 1 | *R. sierrae* | 5/19/2016 | 6/17/2016 | 7/24/2016 | 8/18/2016 |
| Lake G | 19 | 4 | *R. sierrae* | 5/31/2016 | 6/21/2016 | 7/23/2016 | 8/20/2016 |
| Lake H | 10 | 2 | *R. sierrae* | 5/23/2016 | 6/15/2016 | 7/24/2016 | 8/17/2016 |
| Lake I | 8 | 2 | *R. sierrae* | 5/21/2016 | 6/18/2016 | 7/20/2016 | 8/16/2016 |
| Lake J | 18 | 4 | *R. sierrae* | 5/17/2016 | 6/13/2016 | 7/21/2016 | 8/15/2016 |

**Appendix 4**

| Filter Type and Volume Range (mLs) | | | | | | | |
| --- | --- | --- | --- | --- | --- | --- | --- |
| Location | Sampling Occasion 1 | Sampling Occasion 2 | | Sampling Occasion 3 | | Sampling Occasion 4 | |
|  | CN | CN | PES | CN | MCE | CN | MCE |
| Lake F | 600-1000 | 500-1000 | **1000-1250** | 500 | **1000-2000** | 500 | **1000-1240** |
| Lake G | 500-950 | **375-650** | 350-525 | 500 | **1000-2000** | 475-500 | **1000** |
| Lake H | 230-1000 | **255-495** | 240-380 | 500 | **1000-2000** | 350-450 | **1000-1400** |
| Lake I | 500-1000 | 500-1000 | **350-1200** | **1000** | -- | 500 | **1000-2000** |
| Lake J | 500-950 | **300-480** | 325-475 | 200-250 | **225-1000** | 150-425 | **125-1000** |

**Appendix 5**

| Filter Type and Cup with Volume Range (mLs) | | | |
| --- | --- | --- | --- |
| Location | 0.45 µm CN/ Nalgene Cup | 5 µm MCE/ Nalgene Cup | 5 µm PES/ Sterlitech Cup |
| Lake G | 290-400 | **500** | **500** |
| Lake I | 250-495 | **500** | **460-500** |
| Lake J | 200-300 | **500** | **500** |

**Appendix 6**

| Water Temperature (°C) | | | | |
| --- | --- | --- | --- | --- |
| eDNA Protocol Location | Sampling Occasion 1 | Sampling Occasion 2 | Sampling Occasion 3 | Sampling Occasion 4 |
| Stream A | 11.4 - 15.8 | 11.6 - 14.6 | 13.3 - 19.4 | 19.8 - 23.5 |
| Stream B | 7.3 - 8.1 | 8.4 - 9.6 | 8.6 - 10.9 | 9.8 - 10.2 |
| Stream C | 9.5 - 11.9 | 11.3 - 12.3 | 12.7 - 14.2 | 14.3 - 15.8 |
| Stream D | 6.4 - 8.2 | 11.4 - 18.7 | 14.9 - 19.3 | 18.1 - 19.5 |
| Stream E | 11.1 - 12.2 | 11 - 12.6 | 14.2 - 20.4 | 17.8 - 20.1 |
| Lake F | 7.7 | 12.8 | 20.6 | 21.3 |
| Lake G | 20 - 22.2 | 18.5 - 20.6 | 20.7 - 22.3 | 22.1 - 24.6 |
| Lake H | 6.4 - 14 | 8.9 - 13.5 | 18.8 - 22 | 20.6 - 21.8 |
| Lake I | 7.8 - 7.9 | 12.7 - 14.4 | 19.1 - 19.5 | 23.4 - 24.8 |
| Lake J | 9.5 - 12.1 | 17.9 - 22.7 | 17.1 - 22.2 | 19.7 - 24.1 |

**Appendix 7**

| pH | | | | |
| --- | --- | --- | --- | --- |
| eDNA Protocol Location | Sampling Occasion 1 | Sampling Occasion 2 | Sampling Occasion 3 | Sampling Occasion 4 |
| Stream A | 8.4 - 8.7 | 8.5 - 8.8 | 8.18 - 8.7 | 8.4 - 9 |
| Stream B | 7.5 - 8.2 | 7.5 - 8.5 | 7.3 - 8 | 7.8 - 8.3 |
| Stream C | 7.9 - 8.3 | 7.7 - 8.6 | 7.5 - 8.2 | 7.7 - 8.4 |
| Stream D | 8 - 8.22 | 8 - 8.5 | 8.1 - 8.3 | 7.6 - 8.6 |
| Stream E | 8 - 8.8 | 8.2 - 8.6 | 8.4 - 8.7 | 8.6 -8.8 |
| Lake F | 6.4 | 8.4 | 7.4 | 7.4 |
| Lake G | 6.9 - 8.3 | 7.2 - 8.2 | 7 - 7.5 | 7.4 - 8.2 |
| Lake H | 6.9 - 7.8 | 7.2 - 8.4 | 7.7 - 7.9 | 7.2 - 7.9 |
| Lake I | 7 - 8.7 | 7.5 - 7.83 | 8.7 - 9.4 | 7.8 - 8.1 |
| Lake J | 6.3 - 6.8 | 8.1 - 8.9 | 7.6 - 8.6 | 7.5 - 8.1 |

**Appendix 8**

Figure 1.5: Comparing eDNA detection rates across sampling sites in lake protocol locations for different filter types. Detection rates are the number of qPCR replicates with a yellow-legged frog detection/ total number of replicates analyzed All the lakes were occupied by *R. sierrae*. Filter type 1 was the 0.45 µm CN filters and was used across all sampling occasions. On sampling occasion 2, filter type 2 was the 5 µm PES filters. For sampling occasions 3 and 4, filter type 3 was the 5 µm MCE filters.

Figure 1.5: Comparing eDNA detection rates across sampling sites in lake protocol locations for different filter types. Detection rates are the number of qPCR replicates with a yellow-legged frog detection/ total number of replicates analyzed All the lakes were occupied by *R. sierrae*. Filter type 1 was the 0.45 µm CN filters and was used across all sampling occasions. On sampling occasion 2, filter type 2 was the 5 µm PES filters. For sampling occasions 3 and 4, filter type 3 was the 5 µm MCE filters.

Figure 1.5: Comparing eDNA detection rates across sampling sites in lake protocol locations for different filter types. Detection rates are the number of qPCR replicates with a yellow-legged frog detection/ total number of replicates analyzed All the lakes were occupied by *R. sierrae*. Filter type 1 was the 0.45 µm CN filters and was used across all sampling occasions. On sampling occasion 2, filter type 2 was the 5 µm PES filters. For sampling occasions 3 and 4, filter type 3 was the 5 µm MCE filters.

Figure 1.5: Comparing eDNA detection rates across sampling sites in lake protocol locations for different filter types. Detection rates are the number of qPCR replicates with a yellow-legged frog detection/ total number of replicates analyzed All the lakes were occupied by *R. sierrae*. Filter type 1 was the 0.45 µm CN filters and was used across all sampling occasions. On sampling occasion 2, filter type 2 was the 5 µm PES filters. For sampling occasions 3 and 4, filter type 3 was the 5 µm MCE filters.

Figure 1.5: Comparing eDNA detection rates across sampling sites in lake protocol locations for different filter types. Detection rates are the number of qPCR replicates with a yellow-legged frog detection/ total number of replicates analyzed All the lakes were occupied by *R. sierrae*. Filter type 1 was the 0.45 µm CN filters and was used across all sampling occasions. On sampling occasion 2, filter type 2 was the 5 µm PES filters. For sampling occasions 3 and 4, filter type 3 was the 5 µm MCE filters.

Figure 1.5: Comparing eDNA detection rates across sampling sites in lake protocol locations for different filter types. Detection rates are the number of qPCR replicates with a yellow-legged frog detection/ total number of replicates analyzed All the lakes were occupied by *R. sierrae*. Filter type 1 was the 0.45 µm CN filters and was used across all sampling occasions. On sampling occasion 2, filter type 2 was the 5 µm PES filters. For sampling occasions 3 and 4, filter type 3 was the 5 µm MCE filters.

Figure 1.5: Comparing eDNA detection rates across sampling sites in lake protocol locations for different filter types. Detection rates are the number of qPCR replicates with a yellow-legged frog detection/ total number of replicates analyzed All the lakes were occupied by *R. sierrae*. Filter type 1 was the 0.45 µm CN filters and was used across all sampling occasions. On sampling occasion 2, filter type 2 was the 5 µm PES filters. For sampling occasions 3 and 4, filter type 3 was the 5 µm MCE filters.

Figure 1.5: Comparing eDNA detection rates across sampling sites in lake protocol locations for different filter types. Detection rates are the number of qPCR replicates with a yellow-legged frog detection/ total number of replicates analyzed All the lakes were occupied by *R. sierrae*. Filter type 1 was the 0.45 µm CN filters and was used across all sampling occasions. On sampling occasion 2, filter type 2 was the 5 µm PES filters. For sampling occasions 3 and 4, filter type 3 was the 5 µm MCE filters.

Figure 1.5: Comparing eDNA detection rates across sampling sites in lake protocol locations for different filter types. Detection rates are the number of qPCR replicates with a yellow-legged frog detection/ total number of replicates analyzed All the lakes were occupied by *R. sierrae*. Filter type 1 was the 0.45 µm CN filters and was used across all sampling occasions. On sampling occasion 2, filter type 2 was the 5 µm PES filters. For sampling occasions 3 and 4, filter type 3 was the 5 µm MCE filters.

Figure 1.5: Comparing eDNA detection rates across sampling sites in lake protocol locations for different filter types. Detection rates are the number of qPCR replicates with a yellow-legged frog detection/ total number of replicates analyzed All the lakes were occupied by *R. sierrae*. Filter type 1 was the 0.45 µm CN filters and was used across all sampling occasions. On sampling occasion 2, filter type 2 was the 5 µm PES filters. For sampling occasions 3 and 4, filter type 3 was the 5 µm MCE filters.

Figure 1.5: Comparing eDNA detection rates across sampling sites in lake protocol locations for different filter types. Detection rates are the number of qPCR replicates with a yellow-legged frog detection/ total number of replicates analyzed All the lakes were occupied by *R. sierrae*. Filter type 1 was the 0.45 µm CN filters and was used across all sampling occasions. On sampling occasion 2, filter type 2 was the 5 µm PES filters. For sampling occasions 3 and 4, filter type 3 was the 5 µm MCE filters.

Figure 1.5: Comparing eDNA detection rates across sampling sites in lake protocol locations for different filter types. Detection rates are the number of qPCR replicates with a yellow-legged frog detection/ total number of replicates analyzed All the lakes were occupied by *R. sierrae*. Filter type 1 was the 0.45 µm CN filters and was used across all sampling occasions. On sampling occasion 2, filter type 2 was the 5 µm PES filters. For sampling occasions 3 and 4, filter type 3 was the 5 µm MCE filters.

Figure 1.5: Comparing eDNA detection rates across sampling sites in lake protocol locations for different filter types. Detection rates are the number of qPCR replicates with a yellow-legged frog detection/ total number of replicates analyzed All the lakes were occupied by *R. sierrae*. Filter type 1 was the 0.45 µm CN filters and was used across all sampling occasions. On sampling occasion 2, filter type 2 was the 5 µm PES filters. For sampling occasions 3 and 4, filter type 3 was the 5 µm MCE filters.

Figure 1.5: Comparing eDNA detection rates across sampling sites in lake protocol locations for different filter types. Detection rates are the number of qPCR replicates with a yellow-legged frog detection/ total number of replicates analyzed All the lakes were occupied by *R. sierrae*. Filter type 1 was the 0.45 µm CN filters and was used across all sampling occasions. On sampling occasion 2, filter type 2 was the 5 µm PES filters. For sampling occasions 3 and 4, filter type 3 was the 5 µm MCE filters.

Figure 1.5: Comparing eDNA detection rates across sampling sites in lake protocol locations for different filter types. Detection rates are the number of qPCR replicates with a yellow-legged frog detection/ total number of replicates analyzed All the lakes were occupied by *R. sierrae*. Filter type 1 was the 0.45 µm CN filters and was used across all sampling occasions. On sampling occasion 2, filter type 2 was the 5 µm PES filters. For sampling occasions 3 and 4, filter type 3 was the 5 µm MCE filters.

Figure 1.5: Comparing eDNA detection rates across sampling sites in lake protocol locations for different filter types. Detection rates are the number of qPCR replicates with a yellow-legged frog detection/ total number of replicates analyzed All the lakes were occupied by *R. sierrae*. Filter type 1 was the 0.45 µm CN filters and was used across all sampling occasions. On sampling occasion 2, filter type 2 was the 5 µm PES filters. For sampling occasions 3 and 4, filter type 3 was the 5 µm MCE filters.

Figure 1.5: Comparing eDNA detection rates across sampling sites in lake protocol locations for different filter types. Detection rates are the number of qPCR replicates with a yellow-legged frog detection/ total number of replicates analyzed All the lakes were occupied by *R. sierrae*. Filter type 1 was the 0.45 µm CN filters and was used across all sampling occasions. On sampling occasion 2, filter type 2 was the 5 µm PES filters. For sampling occasions 3 and 4, filter type 3 was the 5 µm MCE filters.

Figure 1.5: Comparing eDNA detection rates across sampling sites in lake protocol locations for different filter types. Detection rates are the number of qPCR replicates with a yellow-legged frog detection/ total number of replicates analyzed All the lakes were occupied by *R. sierrae*. Filter type 1 was the 0.45 µm CN filters and was used across all sampling occasions. On sampling occasion 2, filter type 2 was the 5 µm PES filters. For sampling occasions 3 and 4, filter type 3 was the 5 µm MCE filters.

Figure 1.5: Comparing eDNA detection rates across sampling sites in lake protocol locations for different filter types. Detection rates are the number of qPCR replicates with a yellow-legged frog detection/ total number of replicates analyzed All the lakes were occupied by *R. sierrae*. Filter type 1 was the 0.45 µm CN filters and was used across all sampling occasions. On sampling occasion 2, filter type 2 was the 5 µm PES filters. For sampling occasions 3 and 4, filter type 3 was the 5 µm MCE filters.

Figure 1.5: Comparing eDNA detection rates across sampling sites in lake protocol locations for different filter types. Detection rates are the number of qPCR replicates with a yellow-legged frog detection/ total number of replicates analyzed All the lakes were occupied by *R. sierrae*. Filter type 1 was the 0.45 µm CN filters and was used across all sampling occasions. On sampling occasion 2, filter type 2 was the 5 µm PES filters. For sampling occasions 3 and 4, filter type 3 was the 5 µm MCE filters.

Figure 1.5: Comparing eDNA detection rates across sampling sites in lake protocol locations for different filter types. Detection rates are the number of qPCR replicates with a yellow-legged frog detection/ total number of replicates analyzed All the lakes were occupied by *R. sierrae*. Filter type 1 was the 0.45 µm CN filters and was used across all sampling occasions. On sampling occasion 2, filter type 2 was the 5 µm PES filters. For sampling occasions 3 and 4, filter type 3 was the 5 µm MCE filters.

Figure 1.5: Comparing eDNA detection rates across sampling sites in lake protocol locations for different filter types. Detection rates are the number of qPCR replicates with a yellow-legged frog detection/ total number of replicates analyzed All the lakes were occupied by *R. sierrae*. Filter type 1 was the 0.45 µm CN filters and was used across all sampling occasions. On sampling occasion 2, filter type 2 was the 5 µm PES filters. For sampling occasions 3 and 4, filter type 3 was the 5 µm MCE filters.

Figure 1.5: Comparing eDNA detection rates across sampling sites in lake protocol locations for different filter types. Detection rates are the number of qPCR replicates with a yellow-legged frog detection/ total number of replicates analyzed All the lakes were occupied by *R. sierrae*. Filter type 1 was the 0.45 µm CN filters and was used across all sampling occasions. On sampling occasion 2, filter type 2 was the 5 µm PES filters. For sampling occasions 3 and 4, filter type 3 was the 5 µm MCE filters.

Figure 1.5: Comparing eDNA detection rates across sampling sites in lake protocol locations for different filter types. Detection rates are the number of qPCR replicates with a yellow-legged frog detection/ total number of replicates analyzed All the lakes were occupied by *R. sierrae*. Filter type 1 was the 0.45 µm CN filters and was used across all sampling occasions. On sampling occasion 2, filter type 2 was the 5 µm PES filters. For sampling occasions 3 and 4, filter type 3 was the 5 µm MCE filters.

Figure 1.5: Comparing eDNA detection rates across sampling sites in lake protocol locations for different filter types. Detection rates are the number of qPCR replicates with a yellow-legged frog detection/ total number of replicates analyzed All the lakes were occupied by *R. sierrae*. Filter type 1 was the 0.45 µm CN filters and was used across all sampling occasions. On sampling occasion 2, filter type 2 was the 5 µm PES filters. For sampling occasions 3 and 4, filter type 3 was the 5 µm MCE filters.

Figure 1.5: Comparing eDNA detection rates across sampling sites in lake protocol locations for different filter types. Detection rates are the number of qPCR replicates with a yellow-legged frog detection/ total number of replicates analyzed All the lakes were occupied by *R. sierrae*. Filter type 1 was the 0.45 µm CN filters and was used across all sampling occasions. On sampling occasion 2, filter type 2 was the 5 µm PES filters. For sampling occasions 3 and 4, filter type 3 was the 5 µm MCE filters.

Figure 1.5: Comparing eDNA detection rates across sampling sites in lake protocol locations for different filter types. Detection rates are the number of qPCR replicates with a yellow-legged frog detection/ total number of replicates analyzed All the lakes were occupied by *R. sierrae*. Filter type 1 was the 0.45 µm CN filters and was used across all sampling occasions. On sampling occasion 2, filter type 2 was the 5 µm PES filters. For sampling occasions 3 and 4, filter type 3 was the 5 µm MCE filters.

Figure 1.5: Comparing eDNA detection rates across sampling sites in lake protocol locations for different filter types. Detection rates are the number of qPCR replicates with a yellow-legged frog detection/ total number of replicates analyzed All the lakes were occupied by *R. sierrae*. Filter type 1 was the 0.45 µm CN filters and was used across all sampling occasions. On sampling occasion 2, filter type 2 was the 5 µm PES filters. For sampling occasions 3 and 4, filter type 3 was the 5 µm MCE filters.

Figure 1.5: Comparing eDNA detection rates across sampling sites in lake protocol locations for different filter types. Detection rates are the number of qPCR replicates with a yellow-legged frog detection/ total number of replicates analyzed All the lakes were occupied by *R. sierrae*. Filter type 1 was the 0.45 µm CN filters and was used across all sampling occasions. On sampling occasion 2, filter type 2 was the 5 µm PES filters. For sampling occasions 3 and 4, filter type 3 was the 5 µm MCE filters.

Figure 1.5: Comparing eDNA detection rates across sampling sites in lake protocol locations for different filter types. Detection rates are the number of qPCR replicates with a yellow-legged frog detection/ total number of replicates analyzed All the lakes were occupied by *R. sierrae*. Filter type 1 was the 0.45 µm CN filters and was used across all sampling occasions. On sampling occasion 2, filter type 2 was the 5 µm PES filters. For sampling occasions 3 and 4, filter type 3 was the 5 µm MCE filters.

Figure 1.5: Comparing eDNA detection rates across sampling sites in lake protocol locations for different filter types. Detection rates are the number of qPCR replicates with a yellow-legged frog detection/ total number of replicates analyzed All the lakes were occupied by *R. sierrae*. Filter type 1 was the 0.45 µm CN filters and was used across all sampling occasions. On sampling occasion 2, filter type 2 was the 5 µm PES filters. For sampling occasions 3 and 4, filter type 3 was the 5 µm MCE filters.

Figure 1.5: Comparing eDNA detection rates across sampling sites in lake protocol locations for different filter types. Detection rates are the number of qPCR replicates with a yellow-legged frog detection/ total number of replicates analyzed All the lakes were occupied by *R. sierrae*. Filter type 1 was the 0.45 µm CN filters and was used across all sampling occasions. On sampling occasion 2, filter type 2 was the 5 µm PES filters. For sampling occasions 3 and 4, filter type 3 was the 5 µm MCE filters.

Figure 1.5: Comparing eDNA detection rates across sampling sites in lake protocol locations for different filter types. Detection rates are the number of qPCR replicates with a yellow-legged frog detection/ total number of replicates analyzed All the lakes were occupied by *R. sierrae*. Filter type 1 was the 0.45 µm CN filters and was used across all sampling occasions. On sampling occasion 2, filter type 2 was the 5 µm PES filters. For sampling occasions 3 and 4, filter type 3 was the 5 µm MCE filters.

Figure 1.5: Comparing eDNA detection rates across sampling sites in lake protocol locations for different filter types. Detection rates are the number of qPCR replicates with a yellow-legged frog detection/ total number of replicates analyzed All the lakes were occupied by *R. sierrae*. Filter type 1 was the 0.45 µm CN filters and was used across all sampling occasions. On sampling occasion 2, filter type 2 was the 5 µm PES filters. For sampling occasions 3 and 4, filter type 3 was the 5 µm MCE filters.

Figure 1.5: Comparing eDNA detection rates across sampling sites in lake protocol locations for different filter types. Detection rates are the number of qPCR replicates with a yellow-legged frog detection/ total number of replicates analyzed All the lakes were occupied by *R. sierrae*. Filter type 1 was the 0.45 µm CN filters and was used across all sampling occasions. On sampling occasion 2, filter type 2 was the 5 µm PES filters. For sampling occasions 3 and 4, filter type 3 was the 5 µm MCE filters.

Figure 1.5: Comparing eDNA detection rates across sampling sites in lake protocol locations for different filter types. Detection rates are the number of qPCR replicates with a yellow-legged frog detection/ total number of replicates analyzed All the lakes were occupied by *R. sierrae*. Filter type 1 was the 0.45 µm CN filters and was used across all sampling occasions. On sampling occasion 2, filter type 2 was the 5 µm PES filters. For sampling occasions 3 and 4, filter type 3 was the 5 µm MCE filters.

Figure 1.5: Comparing eDNA detection rates across sampling sites in lake protocol locations for different filter types. Detection rates are the number of qPCR replicates with a yellow-legged frog detection/ total number of replicates analyzed All the lakes were occupied by *R. sierrae*. Filter type 1 was the 0.45 µm CN filters and was used across all sampling occasions. On sampling occasion 2, filter type 2 was the 5 µm PES filters. For sampling occasions 3 and 4, filter type 3 was the 5 µm MCE filters.

Figure 1.5: Comparing eDNA detection rates across sampling sites in lake protocol locations for different filter types. Detection rates are the number of qPCR replicates with a yellow-legged frog detection/ total number of replicates analyzed All the lakes were occupied by *R. sierrae*. Filter type 1 was the 0.45 µm CN filters and was used across all sampling occasions. On sampling occasion 2, filter type 2 was the 5 µm PES filters. For sampling occasions 3 and 4, filter type 3 was the 5 µm MCE filters.

Figure 1.5: Comparing eDNA detection rates across sampling sites in lake protocol locations for different filter types. Detection rates are the number of qPCR replicates with a yellow-legged frog detection/ total number of replicates analyzed All the lakes were occupied by *R. sierrae*. Filter type 1 was the 0.45 µm CN filters and was used across all sampling occasions. On sampling occasion 2, filter type 2 was the 5 µm PES filters. For sampling occasions 3 and 4, filter type 3 was the 5 µm MCE filters.

Figure 1.5: Comparing eDNA detection rates across sampling sites in lake protocol locations for different filter types. Detection rates are the number of qPCR replicates with a yellow-legged frog detection/ total number of replicates analyzed All the lakes were occupied by *R. sierrae*. Filter type 1 was the 0.45 µm CN filters and was used across all sampling occasions. On sampling occasion 2, filter type 2 was the 5 µm PES filters. For sampling occasions 3 and 4, filter type 3 was the 5 µm MCE filters.

Figure 1.5: Comparing eDNA detection rates across sampling sites in lake protocol locations for different filter types. Detection rates are the number of qPCR replicates with a yellow-legged frog detection/ total number of replicates analyzed All the lakes were occupied by *R. sierrae*. Filter type 1 was the 0.45 µm CN filters and was used across all sampling occasions. On sampling occasion 2, filter type 2 was the 5 µm PES filters. For sampling occasions 3 and 4, filter type 3 was the 5 µm MCE filters.

Figure 1.5: Comparing eDNA detection rates across sampling sites in lake protocol locations for different filter types. Detection rates are the number of qPCR replicates with a yellow-legged frog detection/ total number of replicates analyzed All the lakes were occupied by *R. sierrae*. Filter type 1 was the 0.45 µm CN filters and was used across all sampling occasions. On sampling occasion 2, filter type 2 was the 5 µm PES filters. For sampling occasions 3 and 4, filter type 3 was the 5 µm MCE filters.

Figure 1.5: Comparing eDNA detection rates across sampling sites in lake protocol locations for different filter types. Detection rates are the number of qPCR replicates with a yellow-legged frog detection/ total number of replicates analyzed All the lakes were occupied by *R. sierrae*. Filter type 1 was the 0.45 µm CN filters and was used across all sampling occasions. On sampling occasion 2, filter type 2 was the 5 µm PES filters. For sampling occasions 3 and 4, filter type 3 was the 5 µm MCE filters.

Figure 1.5: Comparing eDNA detection rates across sampling sites in lake protocol locations for different filter types. Detection rates are the number of qPCR replicates with a yellow-legged frog detection/ total number of replicates analyzed All the lakes were occupied by *R. sierrae*. Filter type 1 was the 0.45 µm CN filters and was used across all sampling occasions. On sampling occasion 2, filter type 2 was the 5 µm PES filters. For sampling occasions 3 and 4, filter type 3 was the 5 µm MCE filters.

Figure 1.5: Comparing eDNA detection rates across sampling sites in lake protocol locations for different filter types. Detection rates are the number of qPCR replicates with a yellow-legged frog detection/ total number of replicates analyzed All the lakes were occupied by *R. sierrae*. Filter type 1 was the 0.45 µm CN filters and was used across all sampling occasions. On sampling occasion 2, filter type 2 was the 5 µm PES filters. For sampling occasions 3 and 4, filter type 3 was the 5 µm MCE filters.

Figure 1.5: Comparing eDNA detection rates across sampling sites in lake protocol locations for different filter types. Detection rates are the number of qPCR replicates with a yellow-legged frog detection/ total number of replicates analyzed All the lakes were occupied by *R. sierrae*. Filter type 1 was the 0.45 µm CN filters and was used across all sampling occasions. On sampling occasion 2, filter type 2 was the 5 µm PES filters. For sampling occasions 3 and 4, filter type 3 was the 5 µm MCE filters.

Figure 1.5: Comparing eDNA detection rates across sampling sites in lake protocol locations for different filter types. Detection rates are the number of qPCR replicates with a yellow-legged frog detection/ total number of replicates analyzed All the lakes were occupied by *R. sierrae*. Filter type 1 was the 0.45 µm CN filters and was used across all sampling occasions. On sampling occasion 2, filter type 2 was the 5 µm PES filters. For sampling occasions 3 and 4, filter type 3 was the 5 µm MCE filters.

Figure 1.5: Comparing eDNA detection rates across sampling sites in lake protocol locations for different filter types. Detection rates are the number of qPCR replicates with a yellow-legged frog detection/ total number of replicates analyzed All the lakes were occupied by *R. sierrae*. Filter type 1 was the 0.45 µm CN filters and was used across all sampling occasions. On sampling occasion 2, filter type 2 was the 5 µm PES filters. For sampling occasions 3 and 4, filter type 3 was the 5 µm MCE filters.

Figure 1.5: Comparing eDNA detection rates across sampling sites in lake protocol locations for different filter types. Detection rates are the number of qPCR replicates with a yellow-legged frog detection/ total number of replicates analyzed All the lakes were occupied by *R. sierrae*. Filter type 1 was the 0.45 µm CN filters and was used across all sampling occasions. On sampling occasion 2, filter type 2 was the 5 µm PES filters. For sampling occasions 3 and 4, filter type 3 was the 5 µm MCE filters.

Figure 1.5: Comparing eDNA detection rates across sampling sites in lake protocol locations for different filter types. Detection rates are the number of qPCR replicates with a yellow-legged frog detection/ total number of replicates analyzed All the lakes were occupied by *R. sierrae*. Filter type 1 was the 0.45 µm CN filters and was used across all sampling occasions. On sampling occasion 2, filter type 2 was the 5 µm PES filters. For sampling occasions 3 and 4, filter type 3 was the 5 µm MCE filters.

Figure 1.5: Comparing eDNA detection rates across sampling sites in lake protocol locations for different filter types. Detection rates are the number of qPCR replicates with a yellow-legged frog detection/ total number of replicates analyzed All the lakes were occupied by *R. sierrae*. Filter type 1 was the 0.45 µm CN filters and was used across all sampling occasions. On sampling occasion 2, filter type 2 was the 5 µm PES filters. For sampling occasions 3 and 4, filter type 3 was the 5 µm MCE filters.

Figure 1.5: Comparing eDNA detection rates across sampling sites in lake protocol locations for different filter types. Detection rates are the number of qPCR replicates with a yellow-legged frog detection/ total number of replicates analyzed All the lakes were occupied by *R. sierrae*. Filter type 1 was the 0.45 µm CN filters and was used across all sampling occasions. On sampling occasion 2, filter type 2 was the 5 µm PES filters. For sampling occasions 3 and 4, filter type 3 was the 5 µm MCE filters.

Figure 1.5: Comparing eDNA detection rates across sampling sites in lake protocol locations for different filter types. Detection rates are the number of qPCR replicates with a yellow-legged frog detection/ total number of replicates analyzed All the lakes were occupied by *R. sierrae*. Filter type 1 was the 0.45 µm CN filters and was used across all sampling occasions. On sampling occasion 2, filter type 2 was the 5 µm PES filters. For sampling occasions 3 and 4, filter type 3 was the 5 µm MCE filters.

Figure 1.5: Comparing eDNA detection rates across sampling sites in lake protocol locations for different filter types. Detection rates are the number of qPCR replicates with a yellow-legged frog detection/ total number of replicates analyzed All the lakes were occupied by *R. sierrae*. Filter type 1 was the 0.45 µm CN filters and was used across all sampling occasions. On sampling occasion 2, filter type 2 was the 5 µm PES filters. For sampling occasions 3 and 4, filter type 3 was the 5 µm MCE filters.

Figure 1.5: Comparing eDNA detection rates across sampling sites in lake protocol locations for different filter types. Detection rates are the number of qPCR replicates with a yellow-legged frog detection/ total number of replicates analyzed All the lakes were occupied by *R. sierrae*. Filter type 1 was the 0.45 µm CN filters and was used across all sampling occasions. On sampling occasion 2, filter type 2 was the 5 µm PES filters. For sampling occasions 3 and 4, filter type 3 was the 5 µm MCE filters.

Figure 1.5: Comparing eDNA detection rates across sampling sites in lake protocol locations for different filter types. Detection rates are the number of qPCR replicates with a yellow-legged frog detection/ total number of replicates analyzed All the lakes were occupied by *R. sierrae*. Filter type 1 was the 0.45 µm CN filters and was used across all sampling occasions. On sampling occasion 2, filter type 2 was the 5 µm PES filters. For sampling occasions 3 and 4, filter type 3 was the 5 µm MCE filters.
